# Aqua­[4-(hydroxy­imino­meth­yl)pyridine-κ*N* ^1^](pyridine-2,6-dicarboxyl­ato-κ^3^ *O* ^2^,*N*,*O* ^6^)copper(II)

**DOI:** 10.1107/S1600536808019673

**Published:** 2008-07-05

**Authors:** E. M. Mutambi

**Affiliations:** a University of Bristol, Bristol, England BS8 1TS, England

## Abstract

In the title compound, [Cu(C_7_H_3_NO_4_)(C_6_H_6_N_2_O)(H_2_O)], the coordination geometry of the Cu^II^ atom can be described as distorted square pyramidal. The basal plane is defined by one N atom and two O atoms from the deprotonated pyridine-2,6-dicarboxyl­ate ligand, and a pyridyl N atom from the 4-pyridyl aldoxime ligand. The apical position is occupied by a water mol­ecule. O—H⋯O hydrogen bonds lead to the formation of a two-dimensional network.

## Related literature

For related literature, see: Blake *et al.* (2002 ▶); Germán-Acacio *et al.* (2007 ▶); Ucar *et al.* (2007 ▶); Xie *et al.* (2004 ▶).
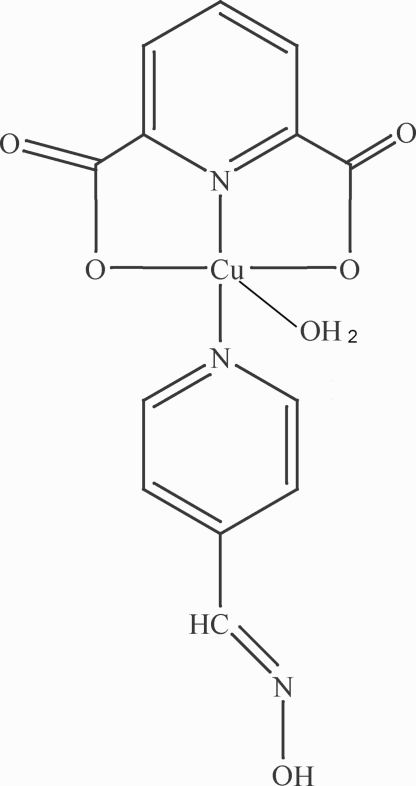


## Experimental

### 

#### Crystal data

[Cu(C_7_H_3_NO_4_)(C_6_H_6_N_2_O)(H_2_O)]
*M* *_r_* = 368.79
Triclinic, 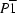

*a* = 6.7826 (2) Å
*b* = 7.1858 (3) Å
*c* = 14.8746 (6) Å
α = 76.154 (2)°
β = 87.152 (1)°
γ = 69.739 (1)°
*V* = 659.91 (4) Å^3^
*Z* = 2
Mo *K*α radiation
μ = 1.69 mm^−1^
*T* = 120 (2) K
0.16 × 0.14 × 0.04 mm

#### Data collection

Bruker–Nonius APEXII CCD diffractometer
Absorption correction: multi-scan (*SADABS*; Sheldrick, 1996 ▶) *T*_min_ = 0.763, *T*_max_ = 0.925
12553 measured reflections
2951 independent reflections
2814 reflections with *I* > 2σ(*I*)
*R*_int_ = 0.055

#### Refinement

*R*[*F* ^2^ > 2σ(*F* ^2^)] = 0.039
*wR*(*F* ^2^) = 0.103
*S* = 1.10
2951 reflections
209 parameters
H-atom parameters constrained
Δρ_max_ = 0.49 e Å^−3^
Δρ_min_ = −0.47 e Å^−3^

### 

Data collection: *APEX2* (Bruker, 2007 ▶); cell refinement: *SAINT* (Bruker, 2007 ▶); data reduction: *SAINT*; program(s) used to solve structure: *SHELXS97* (Sheldrick, 2008 ▶); program(s) used to refine structure: *SHELXL97* (Sheldrick, 2008 ▶); molecular graphics: *SHELXTL* (Sheldrick, 2008 ▶); software used to prepare material for publication: *SHELXTL*.

## Supplementary Material

Crystal structure: contains datablocks I, global. DOI: 10.1107/S1600536808019673/hy2141sup1.cif

Structure factors: contains datablocks I. DOI: 10.1107/S1600536808019673/hy2141Isup2.hkl

Additional supplementary materials:  crystallographic information; 3D view; checkCIF report

## Figures and Tables

**Table:** 

Cu1—N1	1.903 (2)
Cu1—N2	1.957 (2)
Cu1—O2	2.0018 (18)
Cu1—O3	2.0574 (18)
Cu1—O5	2.2273 (18)

**Table:** 

N1—Cu1—N2	168.18 (9)
N1—Cu1—O2	81.66 (8)
N2—Cu1—O2	97.18 (8)
N1—Cu1—O3	79.84 (8)
N2—Cu1—O3	99.02 (8)
O2—Cu1—O3	159.29 (8)
N1—Cu1—O5	91.57 (8)
N2—Cu1—O5	100.24 (8)
O2—Cu1—O5	96.71 (7)
O3—Cu1—O5	93.03 (7)

**Table 2:** Hydrogen-bond geometry (Å, °)

*D*—H⋯*A*	*D*—H	H⋯*A*	*D*⋯*A*	*D*—H⋯*A*
O5—H5*C*⋯O1^i^	0.84	1.93	2.769 (3)	180
O5—H5*B*⋯O4^ii^	0.83	2.07	2.836 (3)	155
O5—H5*B*⋯O6^iii^	0.83	2.51	2.939 (3)	113
O6—H6⋯O3^iv^	0.84	1.89	2.725 (3)	173

Symmetry codes: (i) 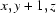; (ii) 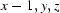; (iii) 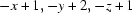; (iv) 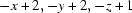.

**Fig. 1.**
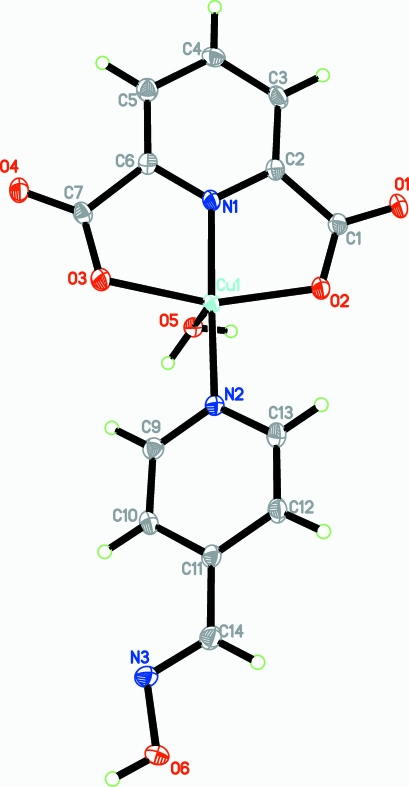
Molecular structure of the title compound, showing the coordination geometry. Displacement ellipsoids are drawn at the 50% probability level.

**Fig. 2.**
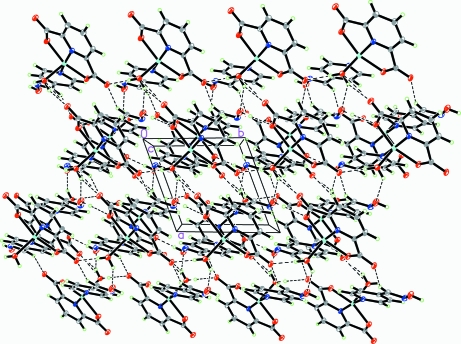
Packing diagram viewed down the c-axis, showing hydrogen bonds (dashed lines).

**Fig. 3.**
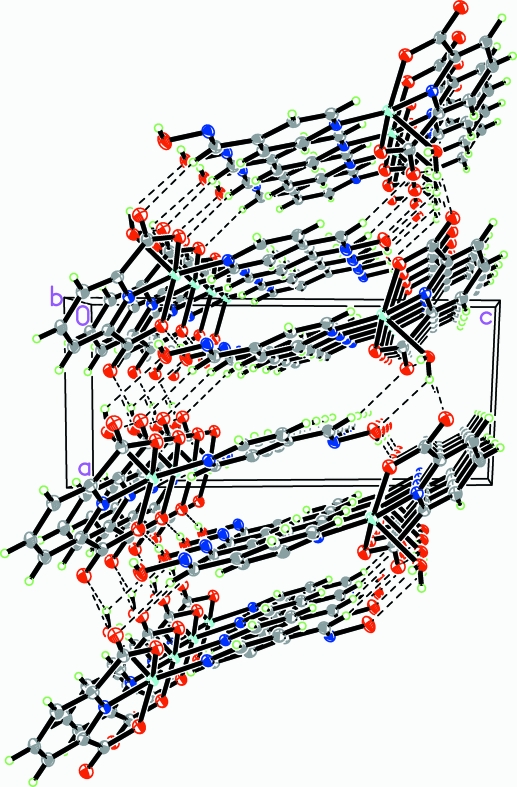
View of a two-dimensional hydrogen-bonded layer along the c-axis. Hydrogen bonds are shown as dashed lines.

## supplementary crystallographic information

### Comment

In the design and synthesis of polymeric complexes, various bridging and chelating ligands have been used extensively. Coordination bonds and hydrogen bonds are the major interactions in these assemblies (Xie *et al.*, 2004). Pyridine-2,6-dicarboxylic acid (H_2_pydc) is an efficient ligand with three coordinating sites. H_2_pydc coordinates with transition metals in different ways to form various coordination geometries. The relative positions of the coordinating atoms (O and N) determine the type of coordination that will be seen in the molecular structure. The interest in this ligand centers on the versatile yet unpredictable manner in which it coordinates to a wide variety of metals due to its rigid and planar nature (Ucar *et al.*, 2007). This paper aims to report one of the rare coordination modes that can be exhibited by copper(II) when coordinated by H_2_pydc, 4-pyridyl aldoxime and H_2_O.

The structure of the title compound is shown in Fig. 1. The molecule is approximately planar and the increased co-planarity is due to the resonance between the pyridine rings, which leads to the formation of square-pyramidal geometry (Fig. 1). The elongated square-pyramidal geometry of the structure (Table 1) is typical of Jahn-Teller-distorted copper(II) (Blake *et al.*, 2002). The structure shows hydrogen-bonding interactions, which enhance the formation of two-dimensional network of the structure (Germán-Acacio *et al.*, 2007). Bond lengths and angles are in the range expected for heteroaromatic-oximes and pryridne dicarboxylates. The hydrogen-bonding interactions are presented in Fig. 2. A l l the hydrogen-bonding donors and acceptors are involved in O—H···O hydrogen bonds (Table 2), which organize the molecules into a two-dimensional network (Fig. 3).

### Experimental

An aqueous solution of Cu(CH_3_COO)_2_.6H_2_O (0.290 g, 1 mmol), KOH (0.220 g, 2 mmol) and H_2_pydc (0.360 g, 2 mmol) in a 1:2:2 molar ratio was refluxed for 2 h and the resultant reaction mixture was reduced to less than 50 ml. After one day, the grown crystals of K_2_[Cu(C_7_H_3_NO_4_)_2_] were filtered out and dried in air. Equimolar amounts of K_2_[Cu(C_7_H_3_NO_4_)_2_] and 4-pyridyl aldoxime were dissolved in water in small vials, respectively, and then mixed together. The solution was left at room temperature in a vapour diffusion setup with ethanol. Blue crystals of the title compound were obtained after 3 weeks.

### Refinement

H atoms bonded to O atoms were located in a difference map and fixed in the refinements with *U*_iso_(H) = 1.5*U*_eq_(O). Other H atoms were positioned geometrically and refined using a riding model, with C—H = 0.95 Å and with *U*_iso_(H) = 1.2*U*_eq_(C).

### Crystal data

**Table:**

[Cu(C_7_H_3_NO_4_)(C_6_H_6_N_2_O)(H_2_O)]	*Z* = 2
*M**_r_* = 368.79	*F*_000_ = 374
Triclinic, *P*1	*D*_x_ = 1.848 Mg m^−^^3^
Hall symbol: -P 1	Mo *K*α radiation λ = 0.71073 Å
*a* = 6.7826 (2) Å	Cell parameters from 18028 reflections
*b* = 7.1858 (3) Å	θ = 2.9–27.5º
*c* = 14.8746 (6) Å	µ = 1.69 mm^−^^1^
α = 76.154 (2)º	*T* = 120 (2) K
β = 87.152 (1)º	Plate, blue
γ = 69.739 (1)º	0.16 × 0.14 × 0.04 mm
*V* = 659.91 (4) Å^3^	

### Data collection

**Table:**

Bruker–Nonius APEXII CCD diffractometer	2951 independent reflections
Radiation source: Bruker-Nonius FR591 rotating anode	2814 reflections with *I* > 2σ(*I*)
Monochromator: 10cm confocal mirrors	*R*_int_ = 0.055
Detector resolution: 4096x4096 pixels / 62x62mm pixels mm^-1^	θ_max_ = 27.4º
*T* = 120(2) K	θ_min_ = 3.1º
φ and ω scans	*h* = 0→8
Absorption correction: multi-scan(SADABS; Sheldrick, 1996)	*k* = −8→9
*T*_min_ = 0.763, *T*_max_ = 0.925	*l* = −18→19
12553 measured reflections	

### Refinement

**Table:**

Refinement on *F*^2^	Secondary atom site location: difference Fourier map
Least-squares matrix: full	Hydrogen site location: inferred from neighbouring sites
*R*[*F*^2^ > 2σ(*F*^2^)] = 0.039	H-atom parameters constrained
*wR*(*F*^2^) = 0.103	*w* = 1/[σ^2^(*F*_o_^2^) + (0.0515*P*)^2^ + 1.382*P*] where *P* = (*F*_o_^2^ + 2*F*_c_^2^)/3
*S* = 1.10	(Δ/σ)_max_ = 0.001
2951 reflections	Δρ_max_ = 0.49 e Å^−^^3^
209 parameters	Δρ_min_ = −0.47 e Å^−^^3^
Primary atom site location: structure-invariant direct methods	Extinction correction: none

### Fractional atomic coordinates and isotropic or equivalent isotropic displacement parameters (Å^2^)

**Table:**

	*x*	*y*	*z*	*U*_iso_*/*U*_eq_	
Cu1	0.89909 (5)	0.56560 (4)	0.26849 (2)	0.01368 (12)	
C1	0.7448 (4)	0.2868 (4)	0.22309 (17)	0.0146 (5)	
C2	0.9262 (4)	0.2935 (4)	0.15912 (17)	0.0140 (5)	
C3	1.0017 (4)	0.1862 (4)	0.09243 (18)	0.0165 (5)	
H3	0.9379	0.0962	0.0797	0.020*	
C4	1.1751 (4)	0.2134 (4)	0.04381 (18)	0.0176 (5)	
H4	1.2321	0.1389	−0.0019	0.021*	
C5	1.2656 (4)	0.3486 (4)	0.06166 (18)	0.0170 (5)	
H5	1.3821	0.3693	0.0281	0.020*	
C6	1.1803 (4)	0.4515 (4)	0.12958 (17)	0.0147 (5)	
C7	1.2478 (4)	0.6059 (4)	0.16230 (17)	0.0145 (5)	
N2	0.8203 (3)	0.6662 (3)	0.38093 (15)	0.0140 (4)	
C9	0.8504 (4)	0.8386 (4)	0.38654 (18)	0.0149 (5)	
H9	0.9058	0.9087	0.3349	0.018*	
C10	0.8047 (4)	0.9181 (4)	0.46359 (18)	0.0152 (5)	
H10	0.8249	1.0422	0.4642	0.018*	
C11	0.7280 (4)	0.8137 (4)	0.54125 (17)	0.0145 (5)	
C12	0.6947 (4)	0.6355 (4)	0.53510 (18)	0.0164 (5)	
H12	0.6404	0.5619	0.5859	0.020*	
C13	0.7416 (4)	0.5671 (4)	0.45432 (18)	0.0161 (5)	
H13	0.7174	0.4465	0.4506	0.019*	
C14	0.6845 (4)	0.8833 (4)	0.62729 (18)	0.0169 (5)	
H14	0.6167	0.8189	0.6759	0.020*	
N1	1.0162 (3)	0.4206 (3)	0.17580 (15)	0.0138 (4)	
N3	0.7389 (3)	1.0317 (4)	0.63636 (15)	0.0172 (4)	
O1	0.6349 (3)	0.1882 (3)	0.21279 (13)	0.0174 (4)	
O2	0.7219 (3)	0.3883 (3)	0.28564 (13)	0.0167 (4)	
O3	1.1531 (3)	0.6594 (3)	0.23461 (13)	0.0175 (4)	
O4	1.3813 (3)	0.6698 (3)	0.12037 (13)	0.0187 (4)	
O5	0.6858 (3)	0.8261 (3)	0.16420 (12)	0.0159 (4)	
H5C	0.6703	0.9360	0.1789	0.024*	
H5B	0.5729	0.8094	0.1586	0.024*	
O6	0.6877 (3)	1.0704 (3)	0.72334 (13)	0.0215 (4)	
H6	0.7376	1.1574	0.7314	0.032*	

### Atomic displacement parameters (Å^2^)

**Table:**

	*U*^11^	*U*^22^	*U*^33^	*U*^12^	*U*^13^	*U*^23^
Cu1	0.01538 (18)	0.01586 (18)	0.01443 (18)	−0.00932 (13)	0.00346 (12)	−0.00687 (12)
C1	0.0162 (12)	0.0134 (11)	0.0143 (11)	−0.0060 (9)	0.0011 (9)	−0.0022 (9)
C2	0.0149 (11)	0.0129 (11)	0.0144 (11)	−0.0061 (9)	0.0004 (9)	−0.0016 (9)
C3	0.0206 (12)	0.0160 (12)	0.0177 (12)	−0.0105 (10)	0.0004 (10)	−0.0063 (10)
C4	0.0225 (13)	0.0182 (12)	0.0156 (12)	−0.0084 (10)	0.0035 (10)	−0.0086 (10)
C5	0.0179 (12)	0.0186 (12)	0.0160 (12)	−0.0079 (10)	0.0005 (9)	−0.0043 (10)
C6	0.0147 (11)	0.0154 (11)	0.0151 (11)	−0.0071 (9)	0.0008 (9)	−0.0028 (9)
C7	0.0166 (12)	0.0152 (11)	0.0139 (11)	−0.0079 (9)	−0.0011 (9)	−0.0039 (9)
N2	0.0138 (10)	0.0160 (10)	0.0149 (10)	−0.0075 (8)	0.0024 (8)	−0.0056 (8)
C9	0.0136 (11)	0.0145 (11)	0.0161 (12)	−0.0045 (9)	0.0008 (9)	−0.0031 (9)
C10	0.0145 (11)	0.0145 (11)	0.0184 (12)	−0.0073 (9)	−0.0003 (9)	−0.0037 (9)
C11	0.0115 (11)	0.0181 (12)	0.0158 (12)	−0.0071 (9)	0.0009 (9)	−0.0050 (9)
C12	0.0168 (12)	0.0194 (12)	0.0167 (12)	−0.0110 (10)	0.0010 (9)	−0.0038 (10)
C13	0.0144 (11)	0.0181 (12)	0.0182 (12)	−0.0087 (10)	0.0004 (9)	−0.0037 (10)
C14	0.0156 (12)	0.0215 (12)	0.0159 (12)	−0.0091 (10)	0.0018 (9)	−0.0048 (10)
N1	0.0157 (10)	0.0154 (10)	0.0142 (10)	−0.0090 (8)	0.0017 (8)	−0.0054 (8)
N3	0.0170 (10)	0.0240 (11)	0.0152 (10)	−0.0099 (9)	0.0035 (8)	−0.0092 (9)
O1	0.0198 (9)	0.0155 (8)	0.0211 (9)	−0.0109 (7)	0.0014 (7)	−0.0051 (7)
O2	0.0199 (9)	0.0189 (9)	0.0168 (9)	−0.0124 (7)	0.0041 (7)	−0.0066 (7)
O3	0.0184 (9)	0.0222 (9)	0.0178 (9)	−0.0119 (7)	0.0030 (7)	−0.0088 (7)
O4	0.0192 (9)	0.0222 (9)	0.0201 (9)	−0.0129 (8)	0.0035 (7)	−0.0069 (7)
O5	0.0147 (8)	0.0166 (8)	0.0188 (9)	−0.0069 (7)	0.0031 (7)	−0.0072 (7)
O6	0.0280 (10)	0.0280 (10)	0.0190 (9)	−0.0173 (9)	0.0085 (8)	−0.0150 (8)

### Geometric parameters (Å, °)

**Table:**

Cu1—N1	1.903 (2)	C7—O3	1.295 (3)
Cu1—N2	1.957 (2)	N2—C9	1.345 (3)
Cu1—O2	2.0018 (18)	N2—C13	1.348 (3)
Cu1—O3	2.0574 (18)	C9—C10	1.375 (4)
Cu1—O5	2.2273 (18)	C9—H9	0.9500
C1—O1	1.229 (3)	C10—C11	1.401 (3)
C1—O2	1.286 (3)	C10—H10	0.9500
C1—C2	1.524 (3)	C11—C12	1.399 (3)
C2—N1	1.334 (3)	C11—C14	1.463 (4)
C2—C3	1.372 (4)	C12—C13	1.387 (4)
C3—C4	1.397 (4)	C12—H12	0.9500
C3—H3	0.9500	C13—H13	0.9500
C4—C5	1.394 (4)	C14—N3	1.280 (3)
C4—H4	0.9500	C14—H14	0.9500
C5—C6	1.380 (4)	N3—O6	1.390 (3)
C5—H5	0.9500	O5—H5C	0.8400
C6—N1	1.335 (3)	O5—H5B	0.8263
C6—C7	1.520 (3)	O6—H6	0.8400
C7—O4	1.231 (3)		
			
N1—Cu1—N2	168.18 (9)	C9—N2—C13	118.4 (2)
N1—Cu1—O2	81.66 (8)	C9—N2—Cu1	118.92 (17)
N2—Cu1—O2	97.18 (8)	C13—N2—Cu1	122.68 (18)
N1—Cu1—O3	79.84 (8)	N2—C9—C10	123.0 (2)
N2—Cu1—O3	99.02 (8)	N2—C9—H9	118.5
O2—Cu1—O3	159.29 (8)	C10—C9—H9	118.5
N1—Cu1—O5	91.57 (8)	C9—C10—C11	119.2 (2)
N2—Cu1—O5	100.24 (8)	C9—C10—H10	120.4
O2—Cu1—O5	96.71 (7)	C11—C10—H10	120.4
O3—Cu1—O5	93.03 (7)	C12—C11—C10	117.8 (2)
O1—C1—O2	125.2 (2)	C12—C11—C14	119.7 (2)
O1—C1—C2	119.9 (2)	C10—C11—C14	122.5 (2)
O2—C1—C2	114.9 (2)	C13—C12—C11	119.5 (2)
N1—C2—C3	120.4 (2)	C13—C12—H12	120.2
N1—C2—C1	111.3 (2)	C11—C12—H12	120.2
C3—C2—C1	128.3 (2)	N2—C13—C12	122.1 (2)
C2—C3—C4	118.0 (2)	N2—C13—H13	119.0
C2—C3—H3	121.0	C12—C13—H13	119.0
C4—C3—H3	121.0	N3—C14—C11	119.3 (2)
C5—C4—C3	120.7 (2)	N3—C14—H14	120.4
C5—C4—H4	119.7	C11—C14—H14	120.4
C3—C4—H4	119.7	C2—N1—C6	122.9 (2)
C6—C5—C4	117.9 (2)	C2—N1—Cu1	117.51 (17)
C6—C5—H5	121.1	C6—N1—Cu1	119.60 (17)
C4—C5—H5	121.1	C14—N3—O6	110.1 (2)
N1—C6—C5	120.2 (2)	C1—O2—Cu1	113.75 (16)
N1—C6—C7	111.4 (2)	C7—O3—Cu1	113.79 (16)
C5—C6—C7	128.5 (2)	Cu1—O5—H5C	109.5
O4—C7—O3	125.6 (2)	Cu1—O5—H5B	110.7
O4—C7—C6	120.1 (2)	H5C—O5—H5B	112.6
O3—C7—C6	114.3 (2)	N3—O6—H6	109.5
			
O1—C1—C2—N1	174.9 (2)	C12—C11—C14—N3	171.6 (2)
O2—C1—C2—N1	−5.5 (3)	C10—C11—C14—N3	−7.7 (4)
O1—C1—C2—C3	−6.4 (4)	C3—C2—N1—C6	0.4 (4)
O2—C1—C2—C3	173.2 (2)	C1—C2—N1—C6	179.2 (2)
N1—C2—C3—C4	0.5 (4)	C3—C2—N1—Cu1	179.26 (19)
C1—C2—C3—C4	−178.1 (2)	C1—C2—N1—Cu1	−1.9 (3)
C2—C3—C4—C5	−1.2 (4)	C5—C6—N1—C2	−0.6 (4)
C3—C4—C5—C6	1.0 (4)	C7—C6—N1—C2	179.3 (2)
C4—C5—C6—N1	−0.1 (4)	C5—C6—N1—Cu1	−179.45 (19)
C4—C5—C6—C7	−179.9 (2)	C7—C6—N1—Cu1	0.4 (3)
N1—C6—C7—O4	−171.9 (2)	N2—Cu1—N1—C2	90.7 (4)
C5—C6—C7—O4	7.9 (4)	O2—Cu1—N1—C2	5.49 (18)
N1—C6—C7—O3	7.7 (3)	O3—Cu1—N1—C2	176.1 (2)
C5—C6—C7—O3	−172.5 (2)	O5—Cu1—N1—C2	−91.07 (19)
N1—Cu1—N2—C9	116.4 (4)	N2—Cu1—N1—C6	−90.4 (4)
O2—Cu1—N2—C9	−160.01 (19)	O2—Cu1—N1—C6	−175.6 (2)
O3—Cu1—N2—C9	32.9 (2)	O3—Cu1—N1—C6	−4.92 (19)
O5—Cu1—N2—C9	−61.82 (19)	O5—Cu1—N1—C6	87.88 (19)
N1—Cu1—N2—C13	−61.9 (5)	C11—C14—N3—O6	−178.7 (2)
O2—Cu1—N2—C13	21.7 (2)	O1—C1—O2—Cu1	−170.6 (2)
O3—Cu1—N2—C13	−145.3 (2)	C2—C1—O2—Cu1	9.9 (3)
O5—Cu1—N2—C13	119.9 (2)	N1—Cu1—O2—C1	−8.63 (17)
C13—N2—C9—C10	0.0 (4)	N2—Cu1—O2—C1	−176.76 (17)
Cu1—N2—C9—C10	−178.33 (19)	O3—Cu1—O2—C1	−35.5 (3)
N2—C9—C10—C11	1.6 (4)	O5—Cu1—O2—C1	81.98 (17)
C9—C10—C11—C12	−2.2 (4)	O4—C7—O3—Cu1	168.1 (2)
C9—C10—C11—C14	177.2 (2)	C6—C7—O3—Cu1	−11.5 (3)
C10—C11—C12—C13	1.1 (4)	N1—Cu1—O3—C7	9.31 (17)
C14—C11—C12—C13	−178.3 (2)	N2—Cu1—O3—C7	177.38 (17)
C9—N2—C13—C12	−1.1 (4)	O2—Cu1—O3—C7	36.3 (3)
Cu1—N2—C13—C12	177.19 (19)	O5—Cu1—O3—C7	−81.74 (18)
C11—C12—C13—N2	0.5 (4)		

### Hydrogen-bond geometry (Å, °)

**Table:**

*D*—H···*A*	*D*—H	H···*A*	*D*···*A*	*D*—H···*A*
O5—H5C···O1^i^	0.84	1.93	2.769 (3)	180
O5—H5B···O4^ii^	0.83	2.07	2.836 (3)	155
O5—H5B···O6^iii^	0.83	2.51	2.939 (3)	113
O6—H6···O3^iv^	0.84	1.89	2.725 (3)	173

Symmetry codes: (i) *x*, *y*+1, *z*; (ii) *x*−1, *y*, *z*; (iii) −*x*+1, −*y*+2, −*z*+1; (iv) −*x*+2, −*y*+2, −*z*+1.
